# Case Report: gastric signet-ring-cell adenocarcinoma in a young adult with tracheoesophageal fistula/esophageal atresia and complex gastrointestinal history

**DOI:** 10.3389/fonc.2026.1774491

**Published:** 2026-05-18

**Authors:** Amira Katrib, Asis Babun, Lauren Wong, Amberly Reynolds, Makoto Nishimura

**Affiliations:** 1 Rocky Vista University, Ivins, UT, United States; 2 Memorial Sloan Kettering Cancer Center, New York, NY, United States

**Keywords:** CARE guidelines, chronic inflammation, dysphagia, endoscopy, esophageal atresia, gastric adenocarcinoma, signet-ring-cell carcinoma, tracheoesophageal fistula

## Abstract

**Introduction:**

Tracheoesophageal fistula with esophageal atresia (TEF/EA) carries long-term gastrointestinal morbidity, including strictures, gastroesophageal reflux disease (GERD), and dysmotility, all of which predispose to chronic inflammation. Gastric signet-ring-cell adenocarcinoma (SRC) is rare in young adults and unusual in the context of congenital foregut anomalies.

**Case presentation:**

A female in her twenties with repaired TEF/EA had progressive dysphagia, recurrent food impactions, and severe distal esophageal stricture requiring frequent dilations. During routine surveillance esophagogastroduodenoscopy (EGD), SRC was incidentally identified. Biopsy handling errors, multiple specimens placed on a single slide labeled “stomach” obscured precise tumor localization.

**Management and outcomes:**

Staging PET-CT showed no metastatic disease. A hereditary cancer panel (ATM, CDH1, CTNNA1, MLH1, MSH2, PMS2, TP53) was negative. Given uncertain tumor location and negative imaging, the patient elected endoscopic surveillance over total gastrectomy. An advanced endoscopist successfully traversed and treated the high-risk stricture, restoring patency and eliminating need for repeat dilations. Over 12 months, serial EGDs demonstrated no residual or recurrent malignancy, and the patient reported marked functional and psychosocial improvement.

**Conclusion:**

This case highlights the interplay of congenital foregut anomalies, chronic inflammation, and SRC development, and underscores the critical importance of meticulous biopsy handling and personalized endoscopic care. Tailored surveillance may be appropriate in select early-stage SRC cases where localization is uncertain and imaging is negative.

## 1 Introduction

Tracheoesophageal fistula with esophageal atresia (TEF/EA) arises from incomplete separation of the trachea and esophagus during embryogenesis and is typically repaired in infancy. Despite surgical correction, survivors frequently experience long-term complications including esophageal strictures, gastroesophageal reflux disease (GERD), dysmotility, and recurrent food impactions leading to chronic mucosal inflammation (1–4).

Gastric adenocarcinoma with signet-ring-cell histology (SRC) is uncommon in young adults and rarely described in TEF/EA populations. Chronic inflammation, repeated epithelial injury, and acid exposure may contribute to carcinogenesis (5–7). Additional contributing factors may include prolonged proton pump inhibitor use and alterations in gastric microbiota (8, 9).

At presentation, the patient was a young adult female with significant functional impairment due to severe dysphagia, requiring dietary restriction and compensatory swallowing maneuvers. Her clinical course was notable for longstanding GERD, recurrent esophageal strictures, and repeated endoscopic dilations, all contributing to chronic mucosal injury. A summarized timeline of the patient’s clinical course, interventions, and surveillance strategy is shown in **Table 1**. She had no history of smoking, alcohol misuse, or known *Helicobacter pylori* infection. There was no known hereditary cancer syndrome based on genetic testing. While traditional risk factors for gastric SRC were absent, her prolonged inflammatory state and mechanical epithelial stress likely played a central role in disease development.

**Table 1:** Clinical timeline of the patient’s disease course.

Date (approx.)	Event	Key details/findings	Impact
Infancy	TEF/EA surgical repair	Survival with residual foregut anomalies	Long-term GI risk
Childhood–Young adulthood	Recurrent food impactions	Frequent endoscopic removal; periodic dilations (q1–2 years)	Persistent dysphagia
Pre-2022	Progressive symptoms	Severe distal stricture; compensatory neck pressure to pass bolus	Functional impairment, psychosocial effects
October 5, 2022	Surveillance EGD with biopsies	Narrowing, GERD-type inflammation; SRC identified	Incidental cancer diagnosis
November 2022	Staging	PET-CT (± contrast): No metastasis	Considered localized disease
Late 2022	Genetics	Negative: ATM, CDH1, CTNNA1, TP53, MLH1, MSH2, PMS2	Non-hereditary suspicion
Late 2022	Management deliberation	Uncertain localization due to biopsy handling; options: gastrectomy vs surveillance	Surveillance elected
Late 2022–2023	Advanced endoscopic stricture traversal	Previously deemed unsafe by others; successful restoration of patency	Resolution of dysphagia/impactions
2023–2024	Serial EGDs q3–6 months	No residual/recurrent malignancy	Functional recovery maintained
Ongoing	Tailored surveillance	Interval endoscopy, clinical exams, and imaging as indicated	Active monitoring

Summary of major clinical events, investigations, and management from infancy through ongoing surveillance. Source: Created by the authors.

This case therefore highlights a non-traditional pathway of carcinogenesis driven primarily by chronic inflammation rather than classical environmental or hereditary risk factors.

What is unique about this case: (i) SRC discovered incidentally during surveillance in a young adult with repaired TEF/EA and severe stricture disease; (ii) biopsy handling errors obscured tumor localization and altered downstream decision-making; (iii) advanced endoscopic traversal of a previously “unsafe” stricture resulted in transformative symptom relief and sustained function; (iv) tailored surveillance was selected over empiric gastrectomy in the context of negative staging and uncertain localization.

## 2 Case description

Patient information (de-identified): Female, twenties, history of TEF/EA repaired in infancy. Longstanding dysphagia, regurgitation, and recurrent food impactions, necessitating esophageal dilations every 1–2 years. Severe distal esophageal stricture caused obstruction even with soft foods; frequent endoscopic food removal for impactions. Patient employed compensatory external neck pressure to facilitate bolus passage, with significant nutritional and psychosocial impact (dietary restriction, avoidance of public eating).

The patient had no documented features of VACTERL association (vertebral, anorectal, cardiac, tracheoesophageal, renal, and limb anomalies) beyond tracheoesophageal fistula/esophageal atresia. No additional congenital anomalies were identified in available medical records.

Clinical findings: Transition to adult GI care revealed severe distal esophageal narrowing and mucosal inflammation consistent with chronic GERD on EGD. Biopsies were obtained.

Diagnostic histopathology: Specimens demonstrated gastric signet-ring-cell adenocarcinoma. Multiple biopsy samples were placed on a single slide labeled “stomach,” preventing confident assignment of lesion location (e.g., cardia vs. body vs. antrum) and complicating management.

Past interventions and outcomes: Repeated esophageal dilations provided transient relief; stricture persisted. Subsequent advanced endoscopic traversal of the high-risk stricture restored durable patency and eliminated the need for further routine dilations.

## 3 Timeline (display item 1)

## 4 Diagnostic assessment, therapeutic interventions, follow-up, and outcomes

### 4.1 Diagnostic assessment

**Figure 1:**
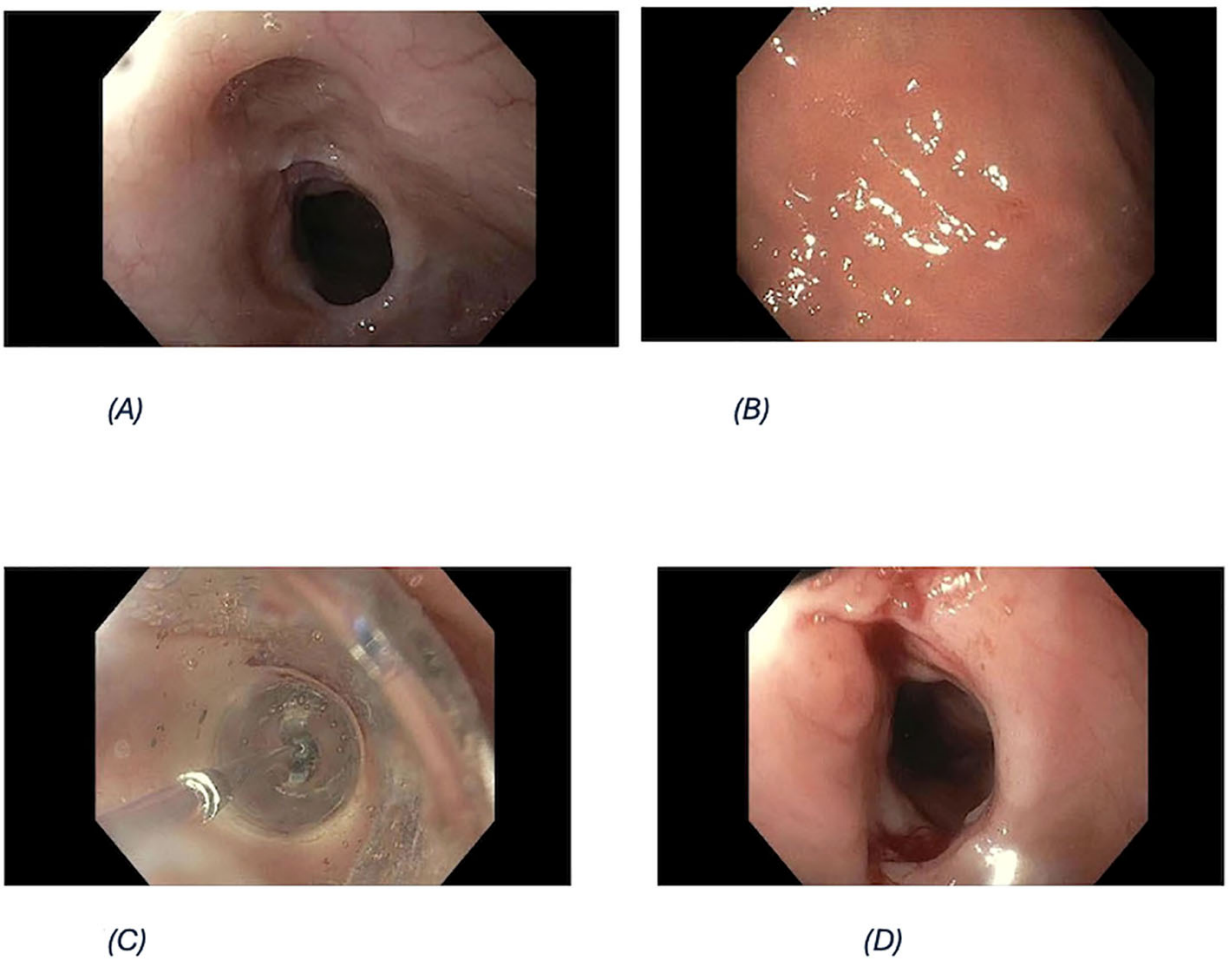
Key endoscopic findings. **(A)** Severe distal esophageal stricture. **(B)** Gastric body biopsy scar. **(C)** Endoscopic dilation of stricture. **(D)** Post-dilation luminal improvement.

- Endoscopy (**Figure 1**): Severe distal esophageal narrowing; mucosal inflammation consistent with chronic GERD.

- Histopathology: Signet-ring-cell gastric adenocarcinoma identified; precise lesion location indeterminate due to combined specimen handling and slide labeling.
- Imaging (staging): PET-CT with and without contrast.

 ○ Low level FDG uptake localizing to the gastroesophageal junction and the proximal gastric cardia; possibly correlates with location of prior dilatations and recently biopsied site of neoplastic disease.

 ○ No definite evidence of FDG-avid neoplastic nodal or metastatic disease.

 ○ FDG focus without identifiable lymph node in the left prevascular mediastinal region; possibly activated brown fat.

- Genetics: Negative for pathogenic variants in CDH1, ATM, CTNNA1, TP53, MLH1, MSH2, PMS2. Maternal family history notable for colon and uterine cancer, suggesting potential but unconfirmed hereditary predisposition.

### 4.2 Therapeutic interventions

**Figure 2:**
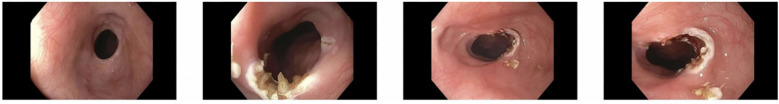
Stenosis transverse with IT knife radial incision.

- Endoscopic management (**Figure 2**): High-risk distal esophageal stricture traversal and treatment by an advanced endoscopist with Japanese techniques, resulting in durable patency and avoidance of repeat routine dilations.
- Oncologic management decision-making: Given negative staging and uncertain localization, options included total gastrectomy versus close surveillance. After multidisciplinary counseling, the patient elected surveillance.

### 4.3 Follow-up and outcomes

- Functional: Resolution of dysphagia and food impactions; discontinuation of compensatory swallowing maneuvers; unrestricted diet; return to public eating without anxiety.
- Oncologic: Serial surveillance EGDs over 12 months revealed no evidence of residual or recurrent malignancy.
- Surveillance protocol: Endoscopy every 3–6 months with interval clinical evaluations; imaging as indicated based on endoscopic findings and symptoms.

## 5 Discussion

This case underscores the association between congenital foregut anomalies and gastric signet ring cell carcinoma in a young adult. Patients with repaired tracheoesophageal fistula and esophageal atresia frequently develop strictures, gastroesophageal reflux, and dysmotility (1–3). These chronic exposures promote inflammation, repeated epithelial injury, and oxidative stress, all of which contribute to carcinogenesis (4). Acid exposure increases mitochondrial superoxide production and lipid peroxidation in gastric epithelium, further supporting malignant transformation (7). Proton pump inhibitor associated hypoacidity may also alter the microbiome and increase gastrin levels, with reported links to preneoplastic changes such as atrophy and intestinal metaplasia (8, 9). These patients should be recognized as a higher risk population and considered for structured, long term endoscopic surveillance.

Signet ring cell carcinoma presents important diagnostic challenges. Early lesions are often subtle and may be difficult to localize, particularly in cases described as “disappearing” carcinoma (10). In this case, pooling of biopsy specimens on a single slide prevented precise localization and directly limited management options. This highlights a correctable systems issue. Biopsy specimens should be submitted in separate, site specific containers with clear labeling, and communication between endoscopists and pathology teams should be standardized. Adherence to these practices is essential to enable accurate mapping, guide endoscopic therapy, and avoid unnecessary surgical intervention.

Management must balance oncologic risk with preservation of function. In young patients with signet ring cell carcinoma, evaluation for hereditary cancer syndromes is essential. Testing should include CDH1 and CTNNA1 for hereditary diffuse gastric cancer, mismatch repair genes for Lynch syndrome, and TP53 for Li Fraumeni syndrome. Negative results in this case reduced the likelihood of an inherited predisposition and supported deviation from routine surgical management. In contrast, identification of pathogenic variants would warrant strong consideration of prophylactic gastrectomy.

When staging studies are negative and lesion localization cannot be established, immediate total gastrectomy should not be reflexively pursued. In carefully selected patients without hereditary risk, a surveillance-based approach is appropriate. This is particularly relevant when surgery would impose substantial long-term nutritional and quality of life consequences, as in this patient whose oral intake had only recently improved following endoscopic therapy. Surveillance should include high quality endoscopy with systematic mapping biopsies and close interval follow up (5).

Gastric signet ring cell carcinoma in patients with repaired tracheoesophageal fistula and esophageal atresia remains rarely reported, and there are no established management guidelines. However, this case supports several key recommendations: recognition of increased risk in this population, strict adherence to biopsy handling protocols, routine evaluation for hereditary syndromes, and consideration of surveillance in selected early-stage presentations. Unlike most reported cases that proceed directly to surgery, this case demonstrates that a non-operative strategy can be safe and effective when guided by careful staging, multidisciplinary input, and structured follow up.

Strengths: Multidisciplinary evaluation; advanced endoscopic expertise resulting in transformative functional improvement; patient-centered decision-making with transparent risk-benefit discussion.

Limitations: Indeterminate lesion localization due to biopsy handling; limited generalizability of surveillance-first strategy; single-case design without long-term (>5-year) outcomes.

Key takeaways: Chronic inflammation in TEF/EA may facilitate rare but aggressive malignancies; meticulous biopsy handling is essential; advanced endoscopy can avert morbidity in complex strictures; surveillance can be appropriate in carefully selected early SRC scenarios.

## 6 Patient perspective

When you read about tracheoesophageal fistula and esophageal atresia, it sounds like something that gets fixed with surgery as a baby and that is the end of it. But nobody talks about what happens after. Nobody tells you that it follows you for the rest of your life, affecting how you eat, where you eat, and how you feel about eating in front of others.

### 6.1 Investigations (summary)

- Esophagogastroduodenoscopy: Severe distal esophageal narrowing; mucosal inflammation consistent with chronic GERD.
- Biopsy results: Signet-ring-cell gastric adenocarcinoma; pooled specimens prevented precise localization.
- PET-CT (± contrast): No evidence of metastatic disease.
- Genetic panel: Negative for pathogenic mutations (CDH1, ATM, CTNNA1, TP53, MLH1, MSH2, PMS2).

### 6.2 Outcome and follow-up

- Post-intervention outcome: Durable restoration of esophageal patency; resolution of dysphagia and impactions; full diet without restrictions; improved psychosocial functioning.
- Surveillance: Serial EGDs over 12 months with no evidence of residual or recurrent malignancy; ongoing tailored protocol with interval exams and endoscopy every 3–6 months.

### 6.3 Learning points/take-home messages

1. In TEF/EA survivors, long-term inflammation, mechanical stress, and tissue remodeling can increase risk for rare gastrointestinal cancers, including SRC.
2. Accurate biopsy sampling, labeling, and handling are critical; pooled specimens can delay localization and restrict management options.
3. Advanced endoscopic interventions, even in anatomies previously deemed “unsafe,” can yield life-changing functional outcomes and reduce procedural burden.
4. In select early SRC cases with negative staging and uncertain localization, individualized surveillance may be an appropriate alternative to immediate radical surgery.

### 6.4 Patient consent for publication

Written informed consent for publication of this case and accompanying details was obtained from the patient (who is also an author).

## Data Availability

The original contributions presented in the study are included in the article/supplementary material. Further inquiries can be directed to the corresponding author.
